# Vasoactive intestinal peptide-expressing interneurons modulate the effect of behavioral state on cortical activity

**DOI:** 10.3389/fncel.2024.1465836

**Published:** 2024-09-12

**Authors:** Ehsan Sabri, Renata Batista-Brito

**Affiliations:** ^1^Dominick P. Purpura Department of Neuroscience, Albert Einstein College of Medicine, Bronx, NY, United States; ^2^Department of Psychiatry and Behavioral Sciences, Albert Einstein College of Medicine, Bronx, NY, United States; ^3^Department of Genetics, Albert Einstein College of Medicine, Bronx, NY, United States

**Keywords:** cortical state, behavioral state, VIP interneurons, spiking synchrony, delta power

## Abstract

Animals live in a complex and changing environment with various degrees of behavioral demands. Behavioral states affect the activity of cortical neurons and the dynamics of neuronal populations, however not much is known about the cortical circuitry behind the modulation of neuronal activity across behavioral states. Here we show that a class of GABAergic inhibitory interneurons that express vasoactive intestinal peptide-expressing interneurons (VIP), namely VIP interneurons, play a key role in the circuits involved in the modulation of cortical activity by behavioral state, as reflected in the mice facial motion. We show that inhibition of VIP interneurons reduces the correlated activity between the behavioral state of the animal and the spiking of individual neurons. We also show that VIP inhibition during the quiet state decreases the synchronous spiking of the neurons but increases delta power and phase locking of spiking to the delta-band activity. Taken together our data show that VIP interneurons modulate the behavioral state-dependency of cortical activity across different time scales.

## 1 Introduction

Behavioral states, also known as arousal states, can change from sleep to quiet wakefulness or to active exploration of the environment (McGinley et al., 2015; Stringer et al., 2019; Vinck et al., 2015). Transitions between alertness, quiet wakefulness, and sleep occur over a period of seconds, and the resultant states are highly correlated with specific rhythmic activity patterns in the neocortex (i.e., oscillatory activity, or cortical states) (Iwańczuk and Guźniczak, 2015; Saper et al., 2010). At the cortical level, these different arousal states are associated with specific oscillatory activities, which are often called “cortical states.” For example, periods of active engagement and attention are associated with desynchronization, or suppression of low-frequency activity (McGinley et al., 2015). Alternately, during periods of slow-wave sleep (SWS), and periods of quiet wakefulness (e.g., tiredness or daydreaming), neocortical rhythms are synchronized, or dominated by low-frequency oscillations (McGinley et al., 2015). Cortical states have been shown to influence information coding, dynamics of neuronal populations, and cognition (Batista-Brito et al., 2017, 2018; Cardin, 2018; McGinley et al., 2015) and a hallmark of many psychiatric disorders is disturbed behavioral states, however despite the importance of cortical states, little is known about the neuronal circuitry that controls their modulation across arousal states. Recent evidence suggests that GABAergic inhibitory neurons (INs) play a key role in the circuits involved in cortical state modulation (Batista-Brito et al., 2017, 2018; Cardin, 2018; McGinley et al., 2015), and INs in the cortex are increasingly linked to schizophrenia and the regulation of cortical states (Akbarian et al., 1996; Batista-Brito et al., 2017; Beasley et al., 2002; Cardin, 2018; Eastwood and Harrison, 2003).

Cortical circuits are composed of excitatory and inhibitory neurons. Inhibitory GABAergic interneurons function to maintain stability within local neural networks. During healthy cortical activity, excitation is balanced by inhibition. GABAergic signaling not only prevents pathology, such as the runaway excitation observed in seizure, but also regulates the dendritic integration of synaptic inputs, influences the precision of spike output, and facilitates the accurate encoding of sensory information. Nevertheless, a major challenge to understanding the contribution of inhibition to brain development and function is the diversity of cortical GABAergic interneurons, which can be subdivided into three distinct classes, namely: (1) fast-spiking cells that express parvalbumin (PV-INs) and target the cell bodies of excitatory pyramidal neurons (PNs), (2) low-threshold spiking cells that express the peptide somatostatin (SOM-INs) and target the distal dendrites of excitatory neurons, and (3) VIP-expressing interneurons (VIP-INs) that predominantly target other interneurons (Tremblay et al., 2016). VIP-INs are important regulators of cortical function (Fu et al., 2014; Karnani et al., 2016b,a; Lee et al., 2013; Pi et al., 2013). These cells receive local and long-range excitatory glutamatergic inputs as well as serotonergic and cholinergic afferents (Tremblay et al., 2016). The coordinated activity of mature VIP-INs is influenced by the behavioral state, as these cells are recruited by arousing events such as the onset of motor activity (Pi et al., 2013; Lee et al., 2013; Askew et al., 2019). VIP-INs of the sensory cortex are activated by the long-range corticocortical inputs from other cortical areas such as the motor cortex, and cholinergic and serotonergic projections from the basal forebrain (Lee et al., 2013). VIP-INs integrate into cortical circuits early in postnatal life (Miyoshi and Fishell, 2011) and genetic alterations of these cells lead to profound alteration of cortical state and sensory responses (Batista-Brito et al., 2017), however, very little is known about how these cells shape the behavioral state dependency of cortical activity.

Here we examined the role of VIP-INs in the function of cortical circuits. We perturbed VIP-IN function by silencing this cell type using chemogenetics. We find that disruption of VIP-INs interferes with the behavioral state-dependent regulation of cortical circuits. VIP inhibition in the quiet state reduces synchronous neuronal spiking while enhancing delta power of the local field potential (LFP), and increasing phase locking of cortical neurons to delta rhythm. Collectively, our findings suggest that VIP interneurons are critical for the regulation of behavioral state-dependent cortical activity across various time scales.

## 2 Results

### 2.1 Comparing the correlation pattern of facial motion and pupil size with mice neuronal spiking in V1

**FIGURE 1:**
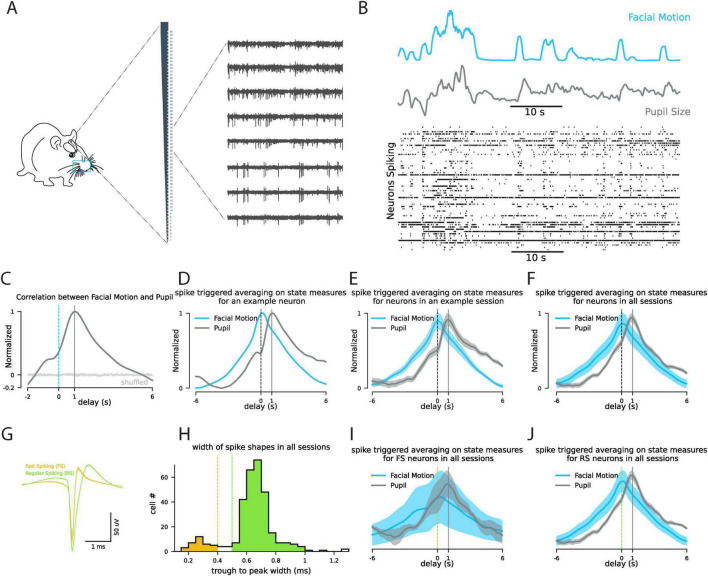
Facial motion has less delay compared to pupil size in following the neuronal spiking in mouse V1. **(A)** Experiment schematic. **(B)** Example window showing simultaneous changes in mouse facial motion energy, and pupil size alongside with the changes in the spiking of the recorded neurons in V1. **(C)** Correlation pattern between the pupil size and the facial motion energy. **(D)** The patterns of spike-triggered averaging on facial motion (blue) and pupil (gray) for an example neuron. **(E)** Same as panel **(D)** for average across all neurons in an example session. **(F)** Same as panel **(D)** for average across all neurons in all the recording sessions from control animals (*n* = 321). **(G)** The average shape of the putative fast spiking neurons (orange, width ¡ 0.4 ms) and putative regular spiking neurons (green, width >0.5 ms). **(H)** The distribution of trough to peak width for all the recorded neurons (*n* = 321). **(I)** Same as panel **(D)** for average across all the putative FS neurons (*n* = 37). **(J)** Same as panel **(D)** for average across all the putative RS neurons (*n* = 273). “delay” refers to the delay between the triggering spikes at 0 and the behavioral signals. “s” refers to seconds.

With advances in the simultaneous recording of neuronal population activity, it has been widely observed that spontaneous fluctuations in population spiking are correlated with behavioral measures of arousal such as locomotion and pupil size (Niell and Stryker, 2010; Vinck et al., 2015). The dynamics of pupil size is broader than locomotion in reflecting spontaneous activity in the cortex because it captures the smaller fluctuations that occur even when the animal is not moving. Changes in mouse facial motion have also been shown to be synchronized with spontaneous population spiking (Figure 1B; Stringer et al., 2019). In our recordings from mice V1, we observe the spiking activity of the majority of neurons is correlated with facial motion and pupil size changes (see an example window of simultaneous single neurons alongside facial motion and pupil size Figure 1B). Here we examine the temporal dynamics of the correlation pattern between pupil size, facial motion, and single-cell spiking to see which one of pupil size and facial motion is more accurate in reflecting the fluctuations of the neuronal spiking in V1. The dynamics of pupil size and facial motion are highly correlated; however, the cross-correlogram of pupil size and facial motion shows that the pupil size is 1 s delayed relative to the facial motion (Figure 1C). To examine the dynamics of correlation between single-neuron spiking and pupil size or facial motion, we calculated the spike-triggered average of each neuron on pupil size and facial motion (see Figure 1D for an example neuron). We observed that although both pupil and facial motion show strong correlations with single-neuron spiking, the pupil is consistently up to 1 s delayed relative to neuronal spiking in V1, while facial motion more synchronously follows neuronal spiking in V1 (Figures 1E, F).

### 2.2 How the suppression of VIP interneurons affect the correlation pattern of V1 neuronal spiking and mice facial motion

**FIGURE 2:**
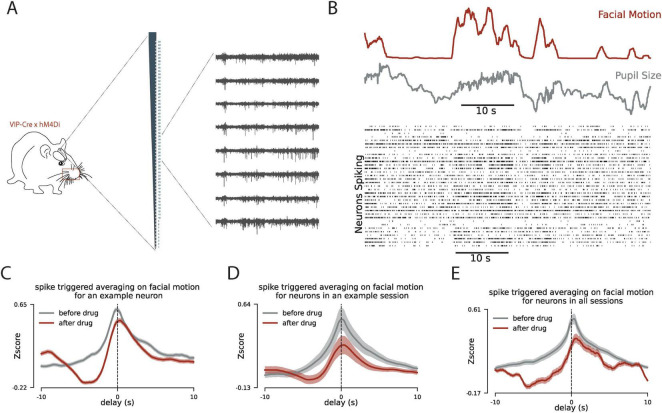
Inhibition of VIP interneurons reduces the correlated dynamics between mouse facial motion and V1 spiking. **(A)** Schematic for the experiment with chemogenetic inhibition of VIP interneurons. **(B)** Same as Figure 1B when the VIP interneurons are suppressed using DREADD. **(C)** The patterns of spike-triggered averaging on the facial motion before (gray) and after the CNO injection (drug) for an example neuron. *y*-axis is normalized relative to the chance level for each pattern. **(D)** Same as panel **(C)** for the average across all neurons in an example session. **(E)** Same as panel **(C)** for the average across all neurons in all recording sessions. In panels **(C–E)** the delay in the *x*-axis quantifies the delay between the spiking activity of neurons and the facial motion.

A large body of evidence suggests that neuromodulators such as Ach play a key role in the synchronized fluctuations in activity in the cortex (Harris and Thiele, 2011; Goard and Dan, 2009; Pi et al., 2013; Lee and Dan, 2012; Lohani et al., 2022). However, the circuitry of the cell types involved in relaying the effect of Ach remains largely unexplored. VIP-expressing interneurons are directly activated by Ach and provide a disinhibitory effect on the local network (Batista-Brito et al., 2017; Lee et al., 2013; Pi et al., 2013), making them a good candidate to play a key role in this circuit. To study the role of VIP interneurons here, we suppress the activity of these cells (Figure 2A) and examine how this changes the pattern of correlation between single-cell spiking and facial motion (Figure 2B). For this, we compared the spike-triggered activity of single neurons and facial motion before and after CNO injection to suppress the activity of VIP interneurons. We observed a decrease in the strength of the correlation between the spiking activity of neurons in V1 and facial motion after VIP suppression (Figures 2C–E). This observation suggests that VIP interneurons are part of the circuit to convey the effect of the behavioral state on the activity of the network. To further characterize the effect of VIP interneuons, we next examine the changes in the network activity after the VIP suppression in this experiment.

### 2.3 During the periods of no facial motion, how does suppression of VIP interneurons change the dynamics of neuronal activity in mice V1?

**FIGURE 3:**
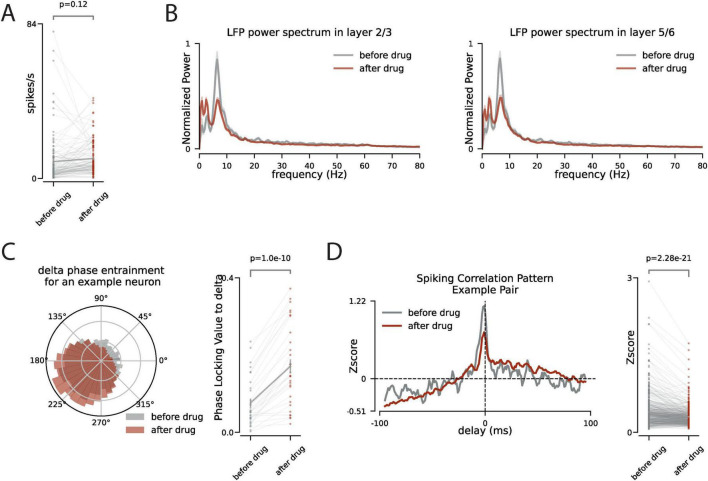
During no facial motion, VIP inhibition increases delta power and phase locking of spiking to the delta but decreases the synchronous spiking of the neurons. **(A)** During the no facial motion periods (stillness) the firing rate of neurons (*n* = 166) is compared before and after the CNO injection. **(B)** The power spectrum of the LFP during the no facial motion periods before (gray) and after (red) CNO injection, shaded area is s.e.m. **(C)** Left panel: the polar distribution of spiking of an example neuron relative to the phase of delta oscillation. Right panel: the change in phase locking value to the delta oscillation for all neurons with significant phase locking to the delta. **(D)** Left panel: The pattern of spiking synchrony between two example neurons before (gray) and after (red) CNO injection. The horizontal dashed line is the chance level. The *y*-axis is the z-score relative chance level. Right panel: The change in peak of the pairwise spiking synchrony before and after the CNO injection across all pairs of neurons that are recorded simultaneously.

With the suppression of VIP interneurons in VIP-Cre x hM4Di mice, the facial motion significantly decreases (*p* < 0.08 across all the recording sessions). Because of that we next focused on the periods of neural activity in which there are no facial movement and compare the network dynamics before and after the suppression of the VIP interneurons. We observe that with the suppression of VIP interneurons there is a slight increase in the firing rate of neurons in V1 (*p* = 0.12, Figure 3A). However, after the VIP inhibition there is a significant change in the spectral components of the LFP across all layers. Most notably, we observe a decrease in the power of alpha-band oscillations and an increase in the power of slower delta-band oscillations (Figure 3B). This spectral shift is also reflected in the coherence between spiking and LFP. We measured the phase locking value between neuronal spiking and delta-band activity and we observed a higher synchrony to delta-band activity after the VIP suppression (Figure 3C). This shift in the network spectral component toward slower rhythms suggests more synchronized spiking across the network. However, by examining the spiking synchrony across neuronal pairs, we observe a decrease in the peak of spiking correlation patterns (Figure 3D) which suggests that VIP suppression modulates the network activity differently across different time scales.

## 3 Discussion

To study the circuitry behind the effect of behavioral state on neural cortical dynamics we focused on the VIP interneurons. VIP interneurons are well studied for their disinhibitory effect on the network through the SST interneurons (Pi et al., 2013). They also receive neuromodulatory input (Tremblay et al., 2016) thus making them a good candidate to play a key role in this circuit. To quantify the network spiking fluctuations that are correlated with the behavioral state, we used spike-triggered averaging to show that facial motion follows the neural dynamics more temporally accurate than pupil size. Next, we used chemogenetics to silence the activity of the VIP interneurons and observed that the correlated dynamics of the neuronal spiking and facial motion is reduced by the suppression of the activity of the VIP interneurons. Finally, we focused on the periods of no facial motion, before and after the VIP interneuron suppression, to examine the changes in the neural activity because of VIP suppression in the absence of behavioral modulation. We observed that delta power in LFP increases after the VIP suppression, and although the firing rate of neurons remains largely unchanged the phase locking of the spiking to the delta oscillation increases. Further analysis of the spiking correlation across neurons showed that fast spiking synchrony between neurons has been reduced which suggests that VIP suppression effects the network differently in different time scales: VIP suppression induces more slow synchrony in the network but reduces fast synchronous spiking across neurons.

Response variability is one of the first observations in the study of cortical sensory processing. For several decades, spike recording in sensory cortices of mammals has puzzled systems neuroscientists about the source of trial-to-trial variability of neurons’ responses to sensory stimulation (Werner and Mountcastle, 1963). This variability has initially been interpreted as noise (Shadlen and Newsome, 1994), however, recently its potential role has been studied in the efficient processing of sensory signals in the cortex (Kohn et al., 2016). It has been posited that changes in baseline activity, meaning the level of neuronal activity (i.e., internal state, cortical state) when the sensory signal arrives in the network, is the main source of variability. What is the source of fluctuations in the baseline activity (aka spontaneous activity)? Previous works by us and others have shown that behavioral measures of arousal (e.g., pupil size and locomotion) could explain (to some extent) sensory response variability, as well as the spontaneous fluctuations of neuronal activity (Figure 1; Vinck et al., 2015; Niell and Stryker, 2010). Simultaneous recordings of thousands of neurons have also shown that spontaneous neural activity fluctuations (i.e., cortical/behavioral state) are shared across the whole brain (Stringer et al., 2019). Taken together, all suggest that response variability in sensory cortices is caused by cortical/behavioral states that are manifested as changes in excitability across the brain.

A large body of evidence suggests that neuromodulators (especially Ach) play a key role in the synchronized fluctuations in activity across the cortex (Harris and Thiele, 2011). However, the circuitry of the cell types involved in relaying the effect of Ach remains largely unexplored. Different types of inhibitory cell types have been shown to be activated during the mice locomotion (Fu et al., 2014; Lee et al., 2013), however, the more subtle changes in the behavioral state like fluctuations in pupil size or mice facial motion are also reflected in the activity of distinct inhibitory cell types (Muñoz et al., 2017). Here we showed that the activity of VIP-interneurons modulates the effect of the behavioral state on the neuronal spiking in V1. This observation fits well with the well-known disinhibitory effect of VIPs on the network through the SST interneurons (Pi et al., 2013). Because SST interneurons are active during the activated cortical state (Muñoz et al., 2017), they are likely to reduce the effect of direct excitation of Ach into the network. This is consistent with the idea that when VIPs are active, they suppress the inhibition from SSTs and so the Ach becomes more effective in boosting the network activity. Our study provides evidence for the role of VIP-Interneurons in the circuitry behind the correlation between neural activity in V1 and behavioral state, however, it remains to show if VIP-Interneurons play a similar role in other cortical regions. In this study, we have suppressed the activity of the VIP interneurons in the hole brain using chemogenetics in the transgenic animals. The short-range connectivity of VIP interneurons suggests that the observed effects on the network are induced by the local VIP interneurons, however, we need further experiments to completely rule out the possibility of effect of VIP interneurons in other regions by local inhibition of VIP interneurons.

## 4 Methods

### 4.1 Animals, headpost Surgery, and treadmill habituation

All animal handling procedures were performed under guidelines approved by the Albert Einstein College of Medicine Institutional Animal Care and Use Committee and federal guide. For the control experiments (1), we handled wild-type male mice (*n* = 5), aged 3–5 months, for 10–15 min daily for 3 days before headpost surgery. On the day of surgery, the mouse was anesthetized with isoflurane and the scalp was cleaned with Betadine solution. After making a midline incision, the scalp was resected to expose the skull. A tungsten wire (50 μm) was then inserted into the right cerebellum through a small hole. This tungsten wire was connected to a gold-plated pin (World Precision Instruments) to serve as the ground/reference connection during electrophysiology recording. The reference pin and a custom 3D printed headpost were affixed to the skull using dental cement. However, the target recording site, left V1, was left free from cement and was merely sealed with vetbond. After surgery, analgesics were administered to help with recovery. Following a 3- to 5-day recovery period after surgery, the mice began treadmill training. Over the next 4 days, we gradually increased the time the mice were head-fixed on the treadmill, continuing until they appeared comfortable and started running without obvious stress. For VIP suppression experiments (2), we crossed Vip-IRES-cre (C57BL/6J) and R26-LSL-Gi-DREADD lines to express the inhibitory DREADD exclusively at VIP interneurons. Similar procedures as described for the wild-type animals were performed on the transgenic animals (*n* = 4).

### 4.2 Electrophysiology and behavioral state monitoring

Approximately 16–20 h before the recording, a small craniotomy was performed over the left V1 under light anesthesia using isoflurane, ensuring the dura remained intact. After craniotomy, the site was sealed with Kwik-Cast (World Precision Instruments), and the animal was allowed to recover in preparation for the recording session the next day. Single-shank 64-channel silicon probes (Sharpened H3, Cambridge NeuroTech) were used for the recordings. The probe was inserted 1200 μm deep from the dura at a rate of 1 μm/s and then retracted by 100 μm for faster stabilization. The broadband signal was recorded using an RHD USB interface board (Intan) at a rate of 20 k Sample/s. For the LFP, the broadband signal from each channel was low-pass filtered (<200 Hz) and downsampled to 2 k Sample/s. To reduce shared high-frequency noise, the signal from each channel was high-pass filtered (>300 Hz) and subtracted by the median of the high-pass filtered signals across the 64 channels at each time point. These median-corrected high-pass filtered signals were then processed to extract spike times using KiloSort (Pachitariu et al., 2024). The KiloSort output was manually curated to discard non-spike patterns. In VIP suppression experiments we injected 5 μg/kg of CNO in the second half of the experiment.

The left half of the mouse face was recorded with the BFLY-PGE-13S2M-CS camera (FLIR) at 30 frames/s under infrared illumination. The recorded frames were synchronized with the electrophysiology signal using the TTL pulses the camera sends out during each frame’s lens opening. The facemap software was used to extract the pupil size and facial motion (Syeda et al., 2022). To track facial movement, a rectangular window containing the whiskers and nose was selected. At this frame rate (30 frames/s) the temporal resolution (∼33 ms) is enough to capture neural dynamics as fast as beta and slower dynamics as we report in Figure 1. To evaluate the correlation dynamics between spiking activity and behavioral measures (pupil size and facial motion), we calculate the average of the behavioral measures in 20 s windows (±10 s) around each detected spike of each cell (Figures 2C–E). In Figure 1, we have zoomed around the delays up to 6 s to better show the difference between dynamics of pupil size and the facial motion.

To estimate the power spectrum using Fast Fourier Transform (FFT), we used the Welsh method implemented in the Python Scipy package. We applied this FFT estimation on non-overlapping windows of 5 s long. The estimated power spectrum was normalized by the total power of the signal and the spectrum was corrected for the 1/f component (Gerster et al., 2022).

## Data Availability

The raw data supporting the conclusions of this article will be made available by the authors, without undue reservation.
